# Identification of microbial DNA in human cancer

**DOI:** 10.1186/1755-8794-2-22

**Published:** 2009-05-08

**Authors:** Christopher G Duncan, Rebecca J Leary, Jimmy Cheng-Ho Lin, Jordan Cummins, Chunhui Di, Carl F Schaefer, Tian-Li Wang, Gregory J Riggins, Jennifer Edwards, Darell Bigner, Levy Kopelovich, Bert Vogelstein, Kenneth W Kinzler, Victor E Velculescu, Hai Yan

**Affiliations:** 1 Preston Robert Tisch Brain Tumor Center, Pediatric Brain Tumor Foundation Institute, Department of Pathology, Duke University Medical Center, Durham, North Carolina 27710, USA; 2 The Ludwig Center for Cancer Genetics and Therapeutics and The Howard Hughes Medical Institute, The Johns Hopkins Kimmel Cancer Center, Baltimore, Maryland 21231, USA; 3 Center for Biomedical Informatics and Information Technology, National Cancer Institute, Rockville, Maryland 20852, USA; 4 Department of Neurosurgery, Johns Hopkins University Medical School, Baltimore, MD 21231, USA; 5 Division of Cancer Prevention, National Cancer Institute, Bethesda, Maryland 20892-7322, USA

## Abstract

**Background:**

Microorganisms have been associated with many types of human diseases; however, a significant number of clinically important microbial pathogens remain to be discovered.

**Methods:**

We have developed a genome-wide approach, called Digital Karyotyping Microbe Identification (DK-MICROBE), to identify genomic DNA of bacteria and viruses in human disease tissues. This method involves the generation of an experimental DNA tag library through Digital Karyotyping (DK) followed by analysis of the tag sequences for the presence of microbial DNA content using a compiled microbial DNA virtual tag library.

**Results:**

To validate this technology and to identify pathogens that may be associated with human cancer pathogenesis, we used DK-MICROBE to determine the presence of microbial DNA in 58 human tumor samples, including brain, ovarian, and colorectal cancers. We detected DNA from Human herpesvirus 6 (HHV-6) in a DK library of a colorectal cancer liver metastasis and in normal tissue from the same patient.

**Conclusion:**

DK-MICROBE can identify previously unknown infectious agents in human tumors, and is now available for further applications for the identification of pathogen DNA in human cancer and other diseases.

## Background

Pathogens are involved in a variety of human diseases. Of particular interest are emerging infections and idiopathic chronic diseases, including cancer [1]. Nevertheless, clinically important microbial pathogens in human disease are likely to be under-recognized [2,3]. Infectious agents have been identified as pathogens in several human tumors, accounting for approximately 20% of human cancers worldwide [4]. However, this proportion accounts for only a few known viral or bacterial agents. Emerging data suggests the role of additional unknown microbes in tumorigenesis [2]. Detection of these infectious agents could provide novel approaches to the prevention, diagnosis and therapy of human cancer.

In the search for the presence of pathogenic DNA in human disease tissues, two kinds of approaches, candidate-based and subtractive, have been previously used. Consensus polymerase chain reaction (PCR) [5-7] and newer DNA microarray-based screening [8-11] methods have been used for candidate-based foreign sequence identification. Candidate screens employing PCR-based techniques have been successfully used for identification and typing of HPV in cervical cancer [12]. DNA microarrays composed of oligonucleotides corresponding to conserved sequences of multiple viruses have been applied to identify a new xenotropic murine leukemia virus-related virus in human prostate tumor cells [8]. Subtractive methods, including representational difference analysis (RDA) [13], computational subtraction [14-16], and digital transcript subtraction [17], have been used to filter sequence data to identify non-human sequences. RDA has been used in the identification of herpesvirus in Kaposi's sarcoma [18], and a method based on long serial analysis of gene expression (LongSAGE) [19,20], called digital transcriptome subtraction (DTS), has been used to identify a new polyomavirus in Merkel cell carcinoma [21].

While these methods have been successful in microbe identification, current techniques have limitations. Candidate-based approaches only confirm the presence of known viruses or bacteria [6]. In addition, varying mechanisms of infection confound the identification of foreign transcript sequences, as the agent may remain in a latent state for years in the host cell without being detected by gene expression methods.

To overcome these limitations, we developed a pathogen discovery approach that applies computational subtraction to Digital Karyotyping (DK). DK identifies and enumerates short sequence tags to provide a comprehensive view of the genomic content of any DNA sample [22,23]. Generally, experimental tags obtained from a DK library are matched to a virtual tag library derived from the human genome. Although the vast majority of tags are identical to the predicted virtual tags, there are inevitably experimental tags which do not match any human sequence. These sequences originate from a number of sources, including unpublished human sequences, tag site polymorphisms, sequencing errors, or foreign DNA sequences that are not present in the normal human genome.

We developed DK-MICROBE to quantitatively evaluate DK sequences that do not match the human genome and which may be derived from microbial genomic DNA. We verified the sensitivity and specificity of this approach by studying Epstein Barr virus (EBV)-infected human lymphoblastoid cells and murine retrovirus infected tumor xenografts. We then applied this technique to analyze brain, colorectal and ovarian tumors for viral and bacterial genome sequences.

## Methods

### Tumor samples

The DK-MICROBE approach was designed for screening of microbial DNA in cancer tissues. Brain tumor tissue samples were obtained from the Preston Robert Tisch Brain Tumor Center Biorepository at Duke University Medical Center by an IRB-approved protocol. Frozen sections were made from each tumor sample and examined by light microscopy by a board-certified neuropathologist to ensure that more than 95% of the section consisted of tumor cells.

Colorectal cancer DNA samples used to make Digital Karyotyping libraries were derived from liver metastases of different patients. Two of the samples were low-passage cell lines established from liver metastasis, while seven samples were immunopurified from liver metastases using the BerEP4 antibody as previously described [24]. Additional samples used for qPCR confirmation were low-passage xenografts established from liver metastases. Normal DNA samples were obtained from peripheral blood or adjacent normal liver.

Five of the ovarian cancer samples are from patients with high-grade ovarian serous carcinoma. Two of the samples are from cell lines purchased through ATCC. All samples were obtained in accordance with the Health Insurance Portability and Accountability Act (HIPAA).

### DK-MICROBE experimental library generation

The initial aspects of performing DK-MICROBE are based on Digital Karyotyping (DK) [25]. Briefly, genomic DNA is cleaved with the mapping enzyme SacI, which has a 6-bp recognition sequence that is predicted to cut genomic DNA into several hundred thousand pieces that are on average < 10 kb. Biotinylated linkers are ligated to the DNA molecules and then digested with the fragmenting enzyme NlaIII, which has a 4-bp recognition site. DNA fragments containing biotinlylated linkers are separated by using streptavidin-coated magnetic beads and are ligated to new linkers containing a class II MmeI site. The fragments are then cleaved by MmeI, releasing 21-bp tags. Isolated tags are self-ligated to form di-tags, PCR-amplified en masse, concatenated, cloned, and sequenced. The experimental tags, adjacent to the NlaIII fragmenting enzyme (CATG) sites closest to SacI mapping enzyme sites, are computationally extracted from sequence data.

We generated 31 brain tumor, 10 colorectal cancer metastasis, and 7 ovarian cancer DK libraries in our laboratories, and analyzed 10 brain libraries from the Cancer Genome Anatomy Project .

### DK-MICROBE microbial virtual tag library

Generation of an extensive and representative microbial virtual tag library is essential for implementing the DK-MICROBE approach. We have assembled a viral and bacterial sequence database, which includes all of the complete bacterial  and viral  genomes of the NCBI Reference Sequence (RefSeq) collection as of May 9, 2008. The NCBI RefSeq database is a curated, non-redundant collection of reference sequences for each organism [26]. Using this database, representative tag sequences were computationally extracted. The resulting tag collection contained 21 bp sequences at each NlaIII site closest to SacI sites for all bacterial or viral genomes in the NCBI RefSeq collection. Comparison of predicted tag sites from microbial and human genomes identified minimal overlap, as only 1,266 tags were present in both reference libraries, many of which correspond to common repetitive elements. This suggests that most 21 bp tags contain sufficient information to distinguish tags as distinctly human or microbial in origin. The tags which match both human and microbial genomes were excluded from the DK-MICROBE virtual tag library for use in further steps of these analyses. The filtering process removed all tags predicted in the human genome sequence (Build 36, March 24, 2008). A total number of 664,413 microbial virtual tags were obtained in this manner. This microbial virtual tag library (Additional files 1, 2 and 3) allowed us to quantitatively identify viral or bacterial genomes present in the analyzed samples by digitally matching experimentally isolated tags to the virtual tags of the reference library.

### DK-MICROBE bioinformatic analyses

Seventeen bp tag sequences (adjacent to NlaIII sites) were extracted and enumerated. These experimental tags were linked to the microbial virtual tag library, and only tags matching microbial sequences perfectly were further considered. All DK tumor libraries were screened for the presence of exogenous DNA from viruses or microorganisms. Foreign genome presence or absence as indicated by DK-MICROBE was validated by PCR and real-time qPCR. Selected positive samples were verified by using direct nucleic acid sequencing.

### DK-MICROBE web interface

We have generated a web site that is part of the Cancer Genome Anatomy Project (CGAP) which allows users to easily upload and analyze experimental DK-MICROBE tags for microbial DNA content. A search feature for tags against microbial genome sequences is available at . Upon uploading a set of 17 base-pair Digital Karyotyping tags, the DK-MICROBE tool searches for exact matches to virtual tags in the genomes of 2,565 bacterial and viral genomes. DK-MICROBE utilizes the microbial virtual tag library described and will be updated to reflect new microbial genome sequences as they become available.

## Results

### Principles of DK-MICROBE

**Figure 1:**
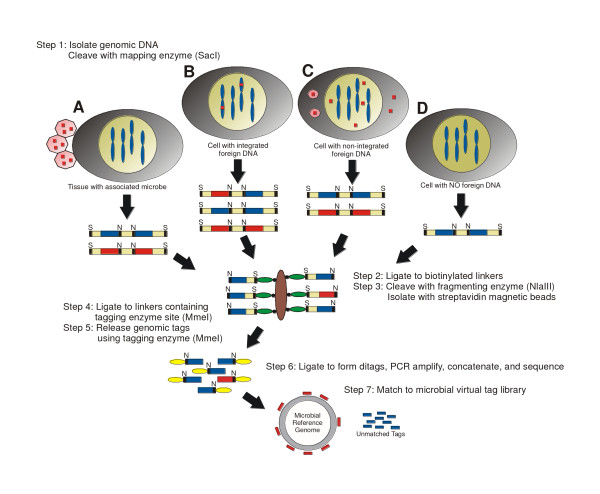
**Schematic of DK-MICROBE**. Tissue of interest may have four potential microbial association states: (A) human cells associated with surrounding microbes, (B) human cells with integrated foreign DNA, (C) human cells with non-integrated foreign DNA, and (D) human cells with no foreign DNA. Sequential restriction digests by SacI (restriction sites indicated by "S") and NlaIII (indicated by "N") result in sequence tags derived from foreign DNA sequences (red boxes), and human sequences (blue boxes). Small ovals represent linkers while large brown ovals represent streptavidin-coated magnetic beads. Experimental tags are matched to microbial virtual tag library, representing all known microbial reference genomes. Unmatched tags include human sequences.

DK-MICROBE provides an *in silico *approach to identify foreign genomic sequences that may be associated with the host cell in several ways (Fig. 1). For example, microbial DNA may be integrated in the host cell genome, may persist as extrachromosomal elements, or may be present in surrounding host tissue. Here, we report an approach developed on the basis of DK and computational subtraction for the identification of microbial sequences in human tissues in any state.

First, the DK-MICROBE approach utilizes the previously described DK protocol for generation of short sequence tags from genomic loci [22,25]. Isolated total genomic DNA is sequentially digested with mapping enzyme SacI, fragmenting enzyme NlaIII, and the type-IIS tagging enzyme MmeI to create 21 bp tags that are concatenated and sequenced to generate a representative genomic profile of the sample. Previous protocols for digital enumeration of DK genomic tags have provided a quantitative measure for copy number alterations of human loci, and have been generally used for identification of cancer gene deletion or amplification [25].

Second, for identification of pathogen genomes in human tissues, we compiled a database comprising all bacterial and viral genomes from existing databases and computationally generated a library that contains microbial virtual tags. The experimental tags that matched the human genome or that had no homology to known microbes are removed such that only exact 21 bp sequences corresponding to the microbial virtual tag library are revealed and used for further analysis.

Finally, BLAST [27] is used to verify tag matches to microbial genomes and to permit extraction of nearby sequences for primer design and PCR based confirmation.

### Probability of detecting microbial DNA using DK-MICROBE

The number of microbial tags to be detected within a given sample by using DK-MICROBE is dependent on the number of virtual tags that a microbial genome contains and the microbial load, which represents the contribution of microbial DNA to the total DNA analyzed. In addition, the degree of sensitivity and confidence for detection of microbial DNA in human tissue samples depends on the total number of tags sequenced. We calculated the probability of detecting the presence of at least one microbial tag in a DK library of a given number of total tags sequenced, for libraries ranging from 50,000 to 2,000,000 tags (Table 1). This analysis showed that detection of a microbe with a large genome (which theoretically contains a large number of virtual tags) can be achieved with minimal sequencing when such microbes are present in virtually all host cells analyzed. For example, viruses or bacteria with an average of 200 tags per cell can be detected with > 99% probability by sequencing 50,000 tags. More challenging is the detection of microbes with small genome size and present with low-level infection. For example, greater than 2 million tags need to be sequenced to detect a virus or bacteria at an average of 2 tags per cell with > 99% probability. Due to the scalable nature of tag sequencing, even a single microbial tag can be detected by DK-MICROBE when a sufficient number of tags are analyzed.

**Table 1:** Probability of detecting microbial virtual tags using DK-MICROBE

Average microbial tag number per cell	Probability of detecting at least one virtual tag given the number of DK tags sequenced (%)^					
	
	50,000	100,000	200,000	500,000	1,000,000	2,000,000
2	4.5	8.7	16.7	36.7	59.9	83.9
20	36.7	59.9	83.9	99	100	100
200	99	100	100	100	100	100

^The probability of detecting at least one microbial tag by sequencing *r *tags is 1 minus the probability of detecting none of the microbial tags among *m *human tags and *n *microbial tags (1-[*m*/(*m*+*n*)]^*r*), where *m *= 2,188,960 (total number of human virtual tags in a diploid genome).

### Testing the presence of EBV tags in lymphoblastoid lines

**Figure 2:**
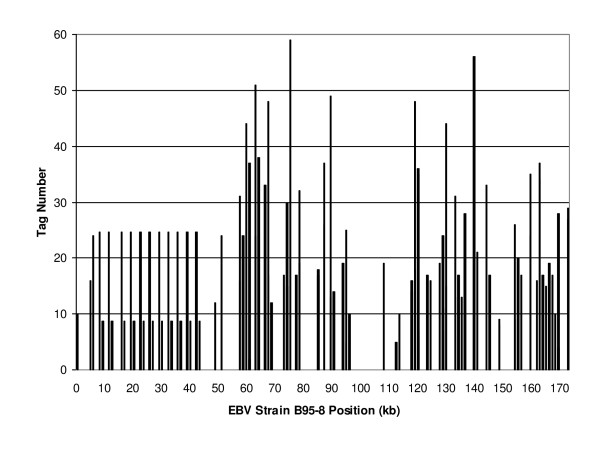
**DK-MICROBE detection of Epstein-Barr viral sequences in normal lymphoblastoid cells**. Experimental tag counts were plotted by position along the EBV strain B95-8 genome. The 366 tags corresponding to EBNA-LP (EBNA leader protein) were distributed evenly among 11 repetitive exons, each containing 2 repeated virtual tags.

To validate the sensitivity and the specificity of the technology, we evaluated human cell lines with known viral infections. We first analyzed a normal lymphoblastoid (NLB) cell line generated through infection with Epstein Barr virus (EBV) [28]. To identify potential viral or bacterial sequences in the NLB sample, 210,752 tags were sequenced and were directly matched with the microbial virtual tag library. Analysis of the NLB library revealed tags which matched both the EBV strain B95-8 and other EBV-related viruses. EBV strain B95-8 contains 94 unique tags and two repetitive tags [29] (Additional file 1). In the NLB cell line, we identified 2,368 experimental tags distributed across the EBV genome (Fig. 2). Among the 2,368 tags, 2,002 (85%) tags matched to the 94 unique virtual tags, with tag numbers ranging from 2 to 59, and 366 (15%) tags matched to the two repetitive tags. Therefore, all 96 of the predicted virtual tags were detected by DK-MICROBE, demonstrating a high level of detection sensitivity. Furthermore, no EBV tags were identified by an analysis of 58 DK libraries derived from tumor samples without any evidence of EBV infection, demonstrating that DK-MICROBE has high specificity.

### Identification of murine retrovirus in xenograft lines

To further investigate the ability of DK-MICROBE to detect naturally occurring exogenous sequences, we examined tumor xenograft samples for the presence of murine retroviruses. It is recognized that xenotropic and ecotropic murine leukemia viruses (MuLVs) are endemic in nude mice and can productively infect transplanted tumor cells [30]. The MuLV genome is approximately 8 kb, giving rise to only 4 virtual tags per genome. The fewer predicted virtual tags in this genome would make detection of these viruses more difficult at lower number of experimental tags than those with larger genomes such as EBV.

**Figure 3:**
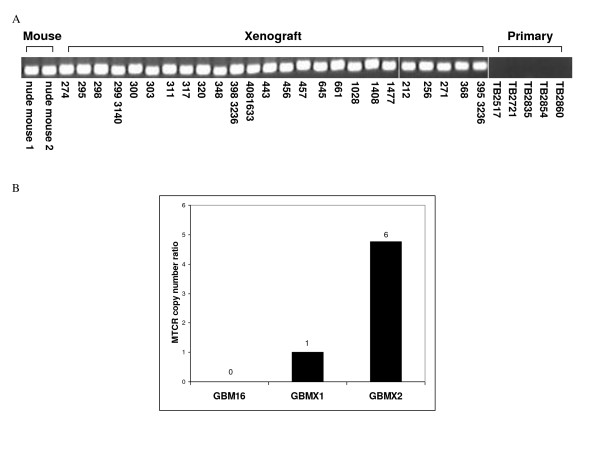
**Identification of murine leukemia virus in xenograft cultures**. A. PCR detection of murine type C retrovirus (MTCR) in brain tumor xenograft cultures and nude mouse, but not primary tumors. B. Comparison between DK-MICROBE tag counts and quantitative real-time PCR (qPCR) data. Amplicons surrounding MTCR DK-MICROBE tags were quantified by Q-PCR in xenografts GBMX1 and GBMX2 and in negative control primary tumor GBM16. DK-MICROBE tag counts are indicated above each bar.

We sequenced 136,308 tags from glioblastoma multiforme (GBM) xenograft GBMX1 and 92,576 tags from GBM xenograft GBMX2 and examined these for the presence of murine retroviruses. DK-MICROBE analysis identified one tag representing the murine type C retrovirus (MTCR) reference genome in sample GBMX1. Six copies of the same tag were identified in GBMX2. PCR analysis and nucleic acid sequencing surrounding the tag confirmed the presence of MTCR (Fig. 3). The tag is located within the envelope gene of MTCR (data not shown). Additional PCR analyses based on the MTCR genome identified MTCR-derived DNA in 100% of 25 brain tumor xenograft samples but not in primary GBMs (Fig. 3A) or cell lines (data not shown). Quantitative PCR (qPCR) was then used to assess relative amounts of viral load between GBMX1, GBMX2, and a primary tumor sample which did not contain murine viral tags by DK-MICROBE (Fig. 3B). We found that the relative viral DNA loading is greater in GBMX2 than in GBMX1 and absent in the primary GBM, consistent with the quantitative results obtained from DK-MICROBE. Similar to the analyses for EBV, MTCR analysis identified no tags from MTCR in 56 DK libraries derived from primary tumor samples.

### Implementation of DK-MICROBE for screening of human cancer samples

To search for the presence of microbial DNA in human tumors, we used DK-MICROBE to analyze 41 brain tumor samples (21 adult GBMs, 2 pediatric GBMs, 3 anaplastic astrocytomas, 1 pediatric astrocytoma, 2 anaplastic oligodendrogliomas, and 12 medulloblastomas), 7 ovarian carcinomas, 3 colorectal carcinomas, and 7 colorectal liver metastases (Table 2). In total, 8.9 million tags were sequenced for the 58 tumor samples, with 63,000 to 243,000 tags sequenced per sample.

**Table 2:** Tumor samples analyzed by DK-MICROBE*

**Tumor Type**	**Sample Name**	**Library Origin**	**# Tags Sequenced**	**Total Pathogen Tags**
Glioblastoma Multiforme	H54OTR	Duke	128056	0
	H80	CGAP	160995	0
	H259	CGAP	199585	1
	H270	CGAP	223570	1
	H336	CGAP	181478	1
	H423	CGAP	170548	4
	H502	CGAP	149766	0
	H542	CGAP	173466	3
	H693	CGAP	210774	1
	H1110	CGAP	194089	4
	H2620	Duke	79026	9
	TB2407	Duke	112358	1
	TB2495	Duke	179070	1
	TB2507	Duke	63386	0
	TB2512	Duke	109182	0
	TB2569	Duke	102768	1
	TB2580	Duke	134658	0
	TB2607	Duke	67042	0
	TB2620	Duke	92576	1
	TB2721	Duke	110354	0
	TB2854	Duke	125282	1

Pediatric Glioblastoma Multiforme	H456	Duke	136308	4
	TB626	Duke	135562	2

Anaplastic Astrocytoma	TB2492	Duke	120466	0
	TB2496	Duke	134908	3
	TB2677	Duke	129710	2

Pediatric Astrocytoma	TB2277	Duke	158294	1

Anaplastic Oligodendroglioma	TB2598	Duke	132894	0
	TB2741	Duke	127798	0

Medulloblastoma	H283	Duke	186836	2
	H341	Duke	205402	0
	H458	Duke	179686	1
	H556	Duke	194696	4
	H581	Duke	185934	1
	TB771	Duke	243126	3
	H876	Duke	157822	0
	TB1339	Duke	194998	1
	TB1389	Duke	177448	1
	TB1961	Duke	183840	2
	TB2226	Duke	192046	1
	MHH1	CGAP	168562	2

Ovarian Carcinoma	463	JHU	120386	0
	505	JHU	125694	1
	713	JHU	99782	0
	1120	JHU	96666	1
	2708	JHU	112912	4
	OVCAR-3	JHU	120242	2
	SKOV-3	JHU	154098	9

Colon Carcinoma	Co84	JHU	142785	1
	Co90	JHU	207016	0
	Co93	JHU	235632	5

Colon Metastasis (Liver)	M10-23	JHU	182482	2
	M11-01	JHU	171898	1
	M12-02	JHU	210906	1
	M12-05	JHU	90780	0
	MJC1	JHU	210888	1
	MJC3	JHU	135264	1
	T4	JHU	118876	4

*Number of tags sequenced and analyzed for each sample are listed by tumor type. Number of tags sequenced and total number of pathogen tags detected are indicated for each sample. Pathogen tag sequence origin is listed in Additional file 4. Abbreviations: CGAP, Cancer Genome Anatomy Project; JHU, Johns Hopkins University.

From all tumor samples, a total of 92 tags were identified as candidate microbial sequences (Additional file 4). Among the 92 tags, eight represented murine type C retroviruses (7 MTCRs and one Rauscher MuLV) in xenograft tumors, all of which we validated by PCR against the MTCR viral genome. Among the remaining tags, only a few have reported associations with human infection, including *Klebsiella pneumonia *[31] and *Burkholderia cenocepacia *[32]. Several tags represent organisms associated with host environments other than the human, such as several plant symbiants and extremophilic species. Selected tags for the most relevant organisms were chosen for validation using PCR-based methods. However, these candidate microbe genomes were not detected in the samples tested, suggesting that infrequent DK tags matching exogenous genomic sequences may represent rare contaminating DNA sequences which are difficult to amplify, or may result from sequencing errors or variation in human genomic sequences that are identical to microbial sequences.

**Figure 4:**
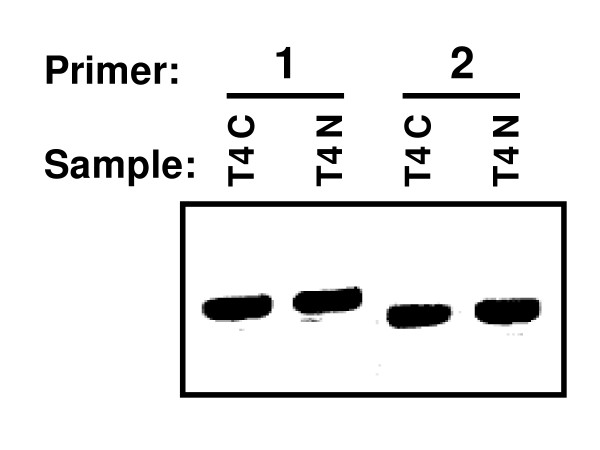
**Identification of HHV-6 in colorectal cancer liver metastasis**. Following identification of HHV-6 by DK-MICROBE, two non-overlapping primer sets were used for PCR confirmation of the presence of HHV-6 DNA in colorectal metastasis T4C and in corresponding normal tissue T4N.

Three of the unique tags identified matched the *Human herpesvirus 6 *(HHV-6) genome. Two of these tags were each observed twice, one was present only once, and all were observed in colorectal liver metastasis T4. PCR analysis using two sets of primers corresponding to the HHV-6 genome confirmed the presence of HHV-6 genomic DNA in this sample, as well as corresponding matched normal tissue from this patient (Fig. 4). Further PCR analyses of additional samples showed that HHV-6 genomic DNA was present in 3 of 7 colorectal cancer samples, and 5 of the 7 matched normal samples.

## Discussion

Microbial pathogens are the etiologic agents of many human diseases and are likely to be under-appreciated in other human illnesses, including cancer. The identification of previously unrecognized pathogens may advance the development of new diagnostic methods and may provide preventative and therapeutic strategies, such as targeted therapy or vaccines to limit tumor-initiating infections.

All current technologies for pathogen identification in human tissues suffer from some common limitations. Varying mechanisms of infection may confound the identification of foreign sequences, as the agent may be present at a very low load in the host cell or function by a transient (hit-and-run) mechanism. In such cases, successful detection of pathogenic sequences is highly reliant on infection stage and cellular composition of the sample. Additionally, these approaches may fail to identify pathogens whose genome composition is unknown. Such methods may also result in false positives, in part because of the similarity of some microbial sequences with the sequence of the host cell. Nevertheless, the overall results of DK-MICROBE indicate a remarkable degree of specificity, even if one assumes that all sequences other than HHV-6 and murine retroviral tags were the result of non-microbial sequences (false-positive rate < 0.001% (81/8,850,672)). As indicated above, the false positives identified by DK-MICROBE may be a result of homology to polymorphic human sequences, sequencing errors, or sequences not present in the current human genome databases.

Understanding the advantages and disadvantages of current platforms for pathogen identification will help guide future use and development of these technologies (Table 3). In comparison to expression-based techniques, methods that use genomic DNA template for pathogen detection, such as DK-MICROBE, have certain advantages. Microbial tag representation for DK-MICROBE is based on genomic length and density of virtual tags within the genome, whereas expression-based technologies are reliant on the number and level of the transcribed genes. Therefore, active expression of microbial genes is not required for detection by DNA-based approaches, and latent microbial genomic fragments can potentially be identified. Additionally, using a genomic DNA-based method allows for utilization of clinical samples not suitable for RNA extraction, such as paraffin-embedded fixed tissue. However, a limitation of using a DNA template is the assumption that the target pathogen has a DNA genome or has been covalently integrated into the human genome. On the other hand, most RNA-based approaches would miss DNA microbial genomes that are not actively transcribed. Additional differences can be noted when considering tag-based platforms against array hybridization methods: While an array hybridization technique is highly versatile in identifying homologous sequences of microorganisms, current microarray design limits identification to a finite number of candidate microbes. On the other hand, a tag-based like method such as DK-MICROBE offers a greater chance of identifying rare microbial associations through extraction of sequences from specific locations in microbial genomes. However, tag length may be a limiting factor when working with novel sequences. With the availability of next generation high throughput sequencing technologies, the number and length of tag sequences would be expected to increase.

**Table 3:** Comparison of available technologies for identification of microbial sequences in human tissues

**(A) Genomic DNA-based platform vs. RNA-based platform**				
**Platform**	**Principle for Analysis**	**Advantages**	**Disadvantages**	**References**

RNA-based	Extraction of sequences from expression library data sets	Detection of actively expressed microbial genes regardless of DNA copy number	No detection of non-transcribed microbial genes	Computational Subtraction [14,16]
		Tags can be generated as long as genes are transcripted	Can miss detection if low microbial load	DTS [17,21]
		Tag numbers are not limited by the genome size	Subject to sequencing errors or tag site polymorphisms matching microbial sequences	Viral Detection DNA Microarray [8]
				RDA [13]

Genomic DNA-based	Extraction of sequences from genomic library data sets	Detection of microbial DNA regardless of expression status of the genes	No detection of non-reverse transcribed RNA	DK-MICROBE
		Potential to detect latent microbial genomic fragments which have been integrated into human genome	Can miss detection if low microbial load	Computational Subtraction [15]
		Utilization of clinical samples not suitable for RNA extraction including paraffin-embedded fixed tissue	Subject to sequencing errors or tag site polymorphisms matching microbial sequences	

**(B) Tag-based vs. array hybridization**				

**Platform**	**Principle for Analysis**	**Advantages**	**Disadvantages**	**References**

Array hybridization	Cross hybridization to homologous sequences of microorganisms on microarray	Currently lower cost	Array designed to target limited number of candidate microbes	Viral Detection DNA Microarray [8-11]
		Method is highly versatile and generally high throughput	Risk of unspecific binding	

Tag-based	Fractional representations through extraction of sequences from specific locations in microbial genomes	Sequence based non biased result	Limited by sequencing costs	DK-MICROBE
		Greater chance to identify rare microbial associations	Subject to sequencing errors, tag site polymorphisms	Computational Subtraction [14-16]
		No physical generation of organism-specific array		DTS [17,21]
		Results can be utilized and referenced for additional human genome analyses		

A. Advantages and disadvantages of using genomic DNA- versus RNA-based platforms. B. Advantages and disadvantages of using tag-based versus array hybridization platforms. Abbreviations: DTS, digital transcript subtraction; RDA, representational difference analysis.

## Conclusion

In this study, we have developed an unbiased approach called DK-MICROBE, for discovery of viral and bacterial associations with human cancer. We validated the sensitivity and specificity of DK-MICROBE in EBV-infected cells, xenografts, and primary cancer tissues. In the normal lymphoblastoid cell line, DK-MICROBE detected all virtual tags of EBV which had been used to transform the cells. In two xenografts, we detected DNA of MuLV, which has a small genome containing only four virtual tags, but did not detect this organism in any of the primary tumors analyzed.

Use of DK-MICROBE identified the presence of HHV-6 DNA in a colorectal cancer metastasis, and subsequent analyses showed that that viral DNA from HHV-6 is present in a subset of colorectal tumors and normal tissues from such individuals. These results demonstrate the ability to discover previously uncharacterized exogenous DNA in primary cancers and tumor metastases using this approach. HHV-6 has been reported to infect a variety of human tissues, and its prevalence in the adult human population is > 85% [33]. Although our data do not directly implicate HHV-6 in colorectal tumorigenesis (since the infection does not appear to be tumor specific), further studies will be needed to determine whether there may be an altered prevalence of colorectal cancer among HHV-6-infected individuals.

Although the studies performed here with DK-MICROBE did not clearly implicate a microbial pathogen in the tumors analyzed, it is clear that this approach has the power to detect exogenous sequences with high sensitivity. Given the association of a variety of viral and bacterial pathogens in the development of human cancer (see [34] for a recent review), it is likely that additional microbial culprits remain to be identified in human tumors. This sensitivity of DK-MICROBE is likely to increase in the future as next-generation sequencing approaches can be used to generate tag libraries of substantially greater sizes. Analysis of larger DK libraries from additional tumor types and samples will increase the probability of detecting unrecognized infectious agents that may play a role in these malignancies. DK-MICROBE may also be useful for analysis of other human diseases and for analysis of microbial populations in various microenvironments. With the complete sequencing of the human genome and further sequence knowledge of additional microbial genomes, the utility, sensitivity, and specificity of DK-MICROBE to detect exogenous sequences will continue to improve.

## Competing interests

Under a licensing agreement between Genzyme Corporation and the Johns Hopkins University, VEV, KWK and BV are entitled to a share of royalties received by the University on sales of products described in this article. The terms of this arrangement are being managed by the Johns Hopkins University in accordance with its conflict of interest policies. Darell D. Bigner is a paid consultant or receives royalties or holds stock in Amgen, Bradmer Pharmaceuticals, and Avant.

## Authors' contributions

CGD performed the EBV and MuLV verification analyses, the DK-MICROBE screening of human cancer samples, and drafted the manuscript. RJL and VEV performed HHV-6 analyses and helped in the drafting of the manuscript. CFS, VEV, JCL and JC created the microbial virtual tag library. HY, DB, and CD contributed brain tumor DK libraries. RJL, JC, BV, KWK, and VEV contributed colorectal DK libraries. TW contributed ovarian DK libraries. GJR and JE contributed CGAP DK libraries. CFS developed the DK-MICROBE web interface. HY, DB, VEV, LK, and BV participated in the design and coordination of the study and helped in the drafting of the manuscript. All authors read and approved the final manuscript.

## Pre-publication history

The pre-publication history for this paper can be accessed here:



## Supplementary Material

Additional file 1 **Table S1.1, Bacterial and Viral Virtual Tag Library**. Library contains 21 bp NlaIII tags closest to SacI sites from indicated bacterial or viral databases (bacterial genomes obtained from ; viral genomes obtained from ) which are not present in the human genome sequence (Build 36, March 24 2008). File 1 of 3. Click here for file

Additional file 2 **Table S1.2, Bacterial and Viral Virtual Tag Library**. Library contains 21 bp NlaIII tags closest to SacI sites from indicated bacterial or viral databases (bacterial genomes obtained from ; viral genomes obtained from ftp://ftp.ncbi.nih.gov/refseq/release/viral/) which are not present in the human genome sequence (Build 36, March 24 2008). File 2 of 3. Click here for file

Additional file 3 **Table S1.3, Bacterial and Viral Virtual Tag Library**. Library contains 21 bp NlaIII tags closest to SacI sites from indicated bacterial or viral databases (bacterial genomes obtained from ; viral genomes obtained from ftp://ftp.ncbi.nih.gov/refseq/release/viral/) which are not present in the human genome sequence (Build 36, March 24 2008). File 3 of 3. Click here for file

Additional file 4 **Table S2, Identification of microbial DNA sequences using DK-MICROBE**. Table of microbial DNA tag sequences observed in the DK-MICROBE human cancer screening. Click here for file
